# The frequency of early age-related macular degeneration and its relationship with dietary pattern in Hunan, China: a cross-sectional study

**DOI:** 10.1186/s12886-022-02549-x

**Published:** 2022-07-27

**Authors:** Yanhui Lin, Ting Peng, Ying Li, Yu Liu

**Affiliations:** 1 Health Management Center, The Third Xiangya Hospital, Central South University, Changsha, Hunan China; 2 Department of Ophthalmology, The Third Xiangya Hospital, Central South University, Street address: No.138,Tongzipo Road,Yuelu District, Hunan 410013 Changsha, China

**Keywords:** Age-related macular degeneration, Dietary pattern, Frequency, Salt intake, Health examination

## Abstract

**Purpose:**

To estimate the frequency of age-related macular degeneration (AMD) among people who underwent health examination in Hunan, China and to determine the relationship between dietary pattern and the risk of AMD.

**Methods:**

The Questionnaire was used to collect dietary data from 56,775 study participants of ≥ 50 years old who underwent health examination at the Department of Health Management, the Third Xiangya Hospital of Central South University between January 2017 and December 2019. The diagnosis of AMD was based on the results of color fundus photography (CFP), spectral-domain optical coherence tomography (OCT) and multispectral imaging (MSI). After excluding participants with incomplete records or other ocular disease that may affect the results of fundus examination, a total of 43,672 study participants were included. The univariate and multivariate logistic regression analyses were used to determine the relationship between dietary pattern and the frequency of AMD.

**Results:**

Among the 43,672 study participants, 1080 (2.5%) had early AMD: the frequencies were 2.6% (*n* = 674) in men and 2.3% (*n* = 406) in women; the frequencies were 1.0% (*n* = 289), 3.6% (*n* = 401), 9.1% (*n* = 390) in 50–59, 60–69, ≥ 70 years old, respectively. And the age-standard frequency was 6.6% over the 60 years old in Hunan China. The high-salt intake increased the risk of early AMD [odds ratio (OR) = 1.61, 95% confidence interval (CI) = 1.54–1.68], whereas the intake of meat decreased the risk (OR = 0.90, 95% CI = 0.81–0.99).

**Conclusion:**

In Hunan China**,** there was a high frequency of early AMD detected through health examination over the 60 years old. And high-salt intake increases the risk of early AMD, whereas intake of meat decreases the risk. Modulating the dietary pattern and reducing the salt intake as an AMD prevention strategy warrant further study.

**Supplementary Information:**

The online version contains supplementary material available at 10.1186/s12886-022-02549-x.

## Background

Age-related macular degeneration (AMD) is the main cause of irreversible blindness among elderly people. Along with the increase of age, the prevalence increases rapidly [1]. AMD not only affects patients’ quality of life but also increases the economic burdens of patients and the society.

Most AMD cases (about 90%) are dry AMD, which presents thickening and structural changes of the Bruch membrane, drusen formed by the lipofuscin accumulation in the retinal pigment epithelia (RPE), and abnormal amount of RPE at early stage, almost without vision impairment. Along with gradual decrease of RPE, it progresses into geographic atrophy (GA), or even choroidal neovascularization (CNV). Blood or blood serum may extravasate through the CNV, and their accumulation may lead to the detachment of neural or pigment epithelia from the retina, presenting obvious vision impairment or loss. Most of current clinical treatments target CNV in wet AMD. The prevention and treatment of dry AMD remain challenging.

Many studies have verified that age, family history, and smoking are risk factors of AMD, and healthy lifestyle may decrease the risk of AMD [2–4]. However, most of these studies were conducted in western countries; big data studies are still lacking in Asian countries, especially in China. China has the largest population among all countries, accounting for more than 1/5 of the world population. Therefore, determine the epidemiologic features of AMD in China and the influence of dietary pattern on this disease may provide reference information for health policy making and health care in China and other countries.

In the present study, we collected dietary data from study participants of ≥ 50 years old who underwent health examination at the Department of Health Management, the Third Xiangya Hospital of Central South University between 2017 and 2019 to analyze frequency of early AMD and its association with dietary pattern.

## Methods

### Study design

**Fig. 1:**
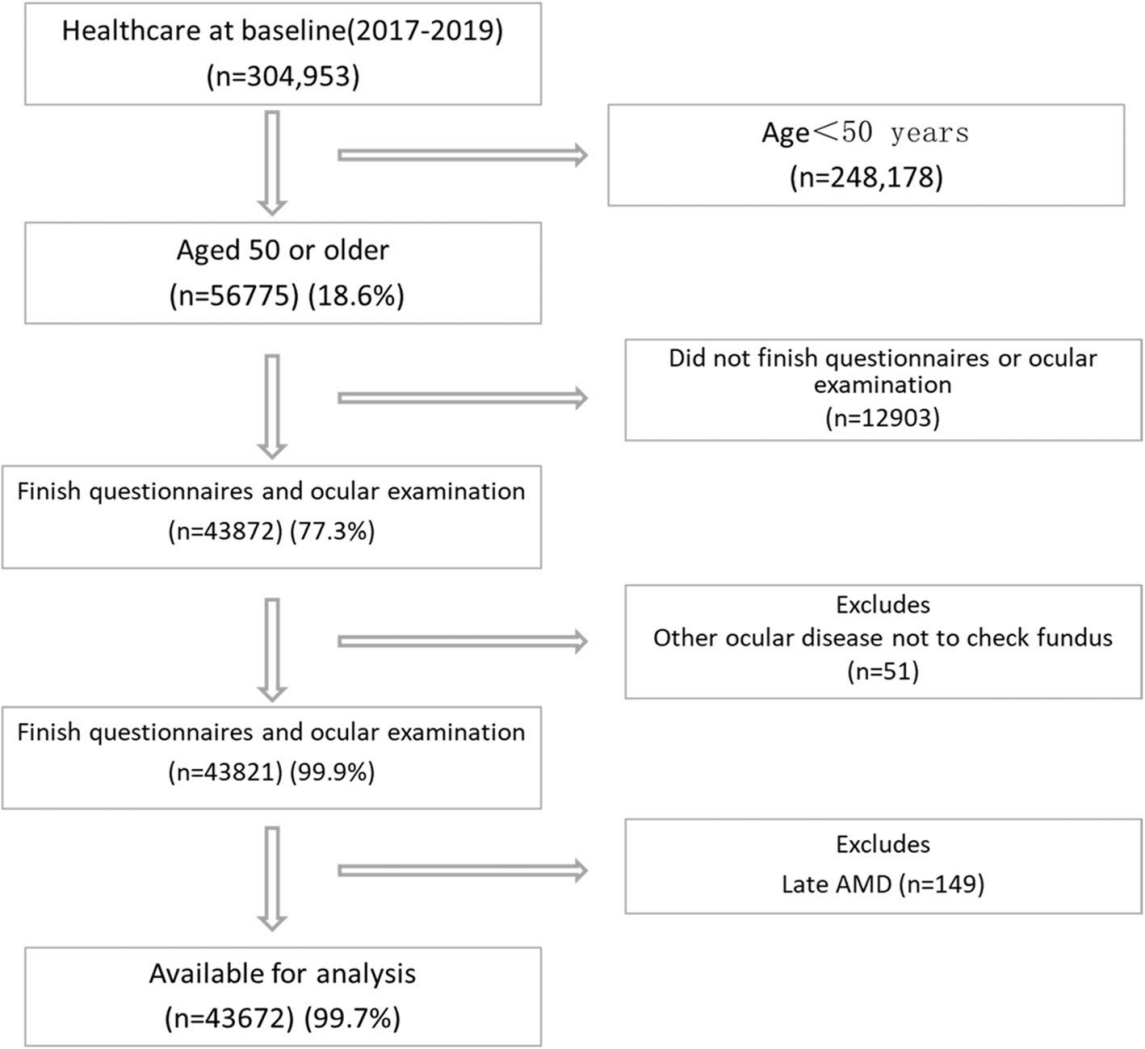
The flow chart of study subject selection in the present study

We selected study participants from people who underwent health examination at the Department of Health Management, the Third Xiangya Hospital of Central South University between January 2017 and December 2019. The selection criteria included age of ≥ 50 years old and the completion of the National Unified Physical Examination Questionnaire and ophthalmologic examinations (Fig. 1).

### The diagnosis criteria of AMD

AMD was diagnosed and graded based on the images of Color fundus photography (CFP) (TRC-NW400, Topcon Healthcare, Beijing China), spectral-domain optical coherence tomography (OCT) (Cirrus HD-OCT, Carl Zeiss Meditec, Inc, California USA) and multispectral imaging (MSI) (ANNIDIS RHA 2020, Annidis Corporation, Ontario Canada) by the diagnostic non-mydriatic imaging. All pictures were centered onto the fovea. OCT analysis was performed on a single horizontal, 6 mm linear scan, and spectralis imaging was performed using the default high-speed mode and the automatic real-time mode.

Each picture was preliminary evaluated as the dichotomous parameter "gradable/ungradable", then gradable images were classified. CFP [5] and MSI [6] images were graded as follows: 0 was graded as normal (no abnormality) findings or normal aging changes, 1 to early AMD (i.e., RPE depigmentation alone, soft drusen alone, or a combination of soft drusen), and 2 was classified as late AMD (i.e., GA or exudative AMD). In addition, if other retinal diseases except AMD are detected, the score was 3. OCT grading images are classified [7–9] according to the following OCT scores: 0 for no abnormal findings, 1 for chorioretinal changes related to early AMD (i.e., RPE abnormalities, drusen), 2 for late AMD (i.e., GA or signs of neovascular AMD), and 3 for other retinal diseases except AMD. In the study, images of 84,372 eyes of 43,672 participants were graded. Pictures of at least one eye per patient can be graded. One skilled technician performed CFP, OCT and MSI examination, and 3 experienced ophthalmologists performed grading analysis on all images.

### Data collection and assessment of diseases

For baseline data collection, all study participants fulfilled the National Unified Physical Examination Questionnaire online [10], which is commonly used in health examination centers in China. It contains items of general lifestyle information, education level, dietary pattern and other habits (e.g., smoking, alcohol drinking, and physical exercises), sleeping, mental health, medical history, and present medication details. The types and duration of occupation, commuting, housework, and physical exercises within the past 12 months were recorded. The weight and height were measured by experienced healthcare staff according to standard protocols: weight was accurate to 0.1 kg, and height was accurate to 0.1 cm. The body mass index (BMI) was calculated by dividing the weight (kg) by the square of height (m^2^). Diabetes mellitus was defined as a fasting glucose level of 11.0 mmol/L or greater or the use of antidiabetic medications. Hypertension was defined as a systolic blood pressure of 140 mm Hg or greater, a diastolic blood pressure of 90 mm Hg or greater, or the use of antihypertensive medications. chronic kidney disease (CKD) was defined according to the medical history of the questionnaire.

### Dietary pattern assessments

Common dietary patterns in China (light food, salt food, or not sure), alcohol drinking, smoking, and some other dietary variables were recorded using the Questionnaire. Light food means diet with less salt, salt food means with salty food and not sure means in the middle of the two kinds. The frequency of these dietary variables within the past 12 months was assessed. The staple food types included primarily refined grain, coarse and refined grain, primarily coarse grain, and not sure. The common foods included domestic animal meat, poultry meat, fish and seafood, animal viscera, eggs, bean products, fresh fruits, fresh vegetables, salted vegetables, and milk products. The frequency of food consumption was classified as four levels: never/rarely, 1–2 days/week, 3–5 days/week, and > 5 days/week.

The frequency and amount of cigarette, alcohol, as well as the frequency and duration of physical exercises were also recorded. The smoking status was classified as never smoking, smoking (more than 1 year, more than 10 cigarettes/day), quit smoking (at least 1 year), and passive smoking (at least 15 min/day, at least once/week). The alcohol drinking status was classified as never drinking, drinking (more than 1 year, more than 1 time/week, more than 50 ml/time), and quit drinking (at least 1 year). The amount of drinking was scaled as 1–2, 3–5, and > 5 days/week. Physical exercise refers to exercise for more than 1 year, more than 1time/week, more than 30 min/time. The frequency of physical exercises was scaled as 0, 1–2, 3–5, and > 5 times/week.Within 1-2 years after baseline data collection, repeated questionnaire assessment was conducted on some (approximately 5%) randomly selected study participants to assess the stability and repeatability of the questionnaire (Supplemental Table S1).

### Salt intake measurement

The 24-h urinary sodium excretion is the golden standard sodium intake measurement because the majority (90%-95%) of ingested sodium was excreted with urine [11]. However, intensive collection of urine samples within 24 h is hard to conduct on participants who underwent health examination. Yang et al. [10] compared the Kawasaki formula, Intersalt formula, and Tanaka formula for the estimation of 24-h urinary sodium excretion using randomly collected urine samples. They found that the Tanaka formula was the most accurate in this estimation. Therefore, the Tanaka formula [12] was used in the present study: 24-h urinary sodium excretion = 23 × 21.98 × (Naspot / Crspot × PrUCr24h) ^0.392^. Naspot = spot urinary sodium (mmol/L); Crspot = spot urinary creatinine (mmol/L); PrUCr24h = 14.89 × weight + 16.14 × height—2.04 × age—2244.45; salt = NaCl = estimated 24-h urinary sodium excretion × 2.55. The urinary sodium and creatinine were measured using potentiometry and enzymatic method, respectively.

### Statistical analysis

Measurement data are presented as mean ± standard deviation (SD) and were compared using the analysis of variance; categorical data were scaled according to the frequency and compared using χ^2^ test. The data from the randomly selected study participants who underwent repeated questionnaire assessment were used to determine the stability and repeatability. The data collected during the first and second questionnaire assessments were paired for comparison. Multivariable logistic stepwise regression analysis was used to calculate odds ratios (ORs) and 95% confidence intervals (CIs) of dietary factors for early AMD. To reduce interrelationship between variables, risk factors with significant differences were analysis by multi-collinear. All statistical analysis were performed using the SPSS18.0 statistical software for windows. *p* < 0.05 was considered significant.

## Results

### Epidemiologic features of early AMD

Between January 2017 and December 2019, a total of 304,953 study participants had undergone health examination at the Department of Health Management, the Third Xiangya Hospital of Central South University. Among 43,821 study participants, 149(0.34%) had late AMD, the rest 43,672 participants, including 1080(2.5%) early AMD and 42,592 participants without signs of AMD, were eligible to be selected as study participants. The mean age was higher in participants with early AMD than in those without early AMD (66.2 ± 0.3 vs. 58.2 ± 0.0, *P* < 0.001). As shown in Table 1, the frequency of early AMD was 2.6% in men and 2.3% in women. It was 1.0% in participants of 50–59 years old, 3.6% in participants of 60–69 years old, and 9.1% in participants of ≥ 70 years old, suggesting that the incidence of early AMD is increasing with aging.

**Table 1:** The associations of early AMD with lifestyle and dietary characteristics of 43,672 participants of ≥ 50 years old who underwent health examination during 2017–2019 (* *p* < 0.05)

Variable	Whole cohort	Early AMD		*P* value
		Presence	Absence	
Total [cases (%)]	43,672	1080 (2.5)	42,592 (97.5)	
Gender[cases (%)]				0.000^*^
Men	26,350 (60.3)	674 (2.6)	25,676 (97.4)	
Women	17,322(39.7)	406 (2.3)	16,916 (97.7)	
Age group [cases (%)]				
50–59 years	28,280(64.8)	289 (1.0)	27,991 (99.0)	
60–69 years	11,103(25.4)	401 (3.6)	10,702 (96.4)	0.000^*^ (vs. 50–59 years group)
≥ 70 years	4289(9.8)	390 (9.1)	3899 (90.9)	0.000^*^ (vs. 60–69 years group)
Waist-to-hip ratio (mean ± SD)	0.91 ± 1.26	0.91 ± 0.00	0.91 ± 0.01	0.223
BMI (mean ± SD)	24.44 ± 2.96	24.65 ± 0.97	24.44 ± 0.15	0.023^*^
Leukocyte count (*10^9^)(mean ± SD)	6.29 ± 2.02	6.30 ± 0.05	6.29 ± 0.01	0.817
Lymphocyte count (*10^12^) (mean ± SD)	2.02 ± 0.67	1.97 ± 0.02	2.02 ± 0.00	0.009^*^
Neotrophil count (*10^9^) (mean ± SD)	3.70 ± 1.46	3.76 ± 0.37	3.79 ± 0.01	0.157
Eosinophil count (*10^9^) (mean ± SD)	0.16 ± 0.16	0.15 ± 0.00	0.16 ± 0.00	0.198
Basophil count (*10^9^) (mean ± SD)	0.03 ± 0.02	0.03 ± 0.00	0.03 ± 0.00	0.611
FBS (mmol/l) (mean ± SD)	5.94 ± 1.59	6.09 ± 0.05	5.94 ± 0.01	0.003^*^
Cholesterol (mmol/l) (mean ± SD)	5.19 ± 1.00	5.07 ± 0.03	5.20 ± 0.00	< 0.001^*^
Triglyceride (mmol/l) (mean ± SD)	1.84 ± 1.57	1.67 ± 0.04	1.85 ± 0.01	< 0.001^*^
HDL (mmol/l) (mean ± SD)	1.37 ± 0.31	1.37 ± 0.01	1.37 ± 0.00	0.323
LDL (mmol/l) (mean ± SD)	2.99 ± 0.87	2.93 ± 0.027	3.00 ± 0.00	0.017^*^
Salt intake (g/d) (mean ± SD)	8.41 ± 1.20	9.26 ± 0.56	8.39 ± 0.68	< 0.001^*^
Education level [cases (%)]	43,672			0.018^*^
Primary school and below	8658(19.8)	264 (3.0)	8394 (97.0)	
Middle/high school	11,149(25.5)	267 (2.4)	10,882 (97.6)	
College	20,421(46.8)	490 (2.4)	19,931 (97.6)	
Postgraduate and above	3444(7.9)	59 (1.7)	3385 (98.3)	
Dietary pattern [cases (%)]	43,672			0.003^*^
Light food	5463(12.5)	118 (2.2)	5345 (97.8)	
Salt food	14,074(32.2)	336 (2.4)	13,738 (97.6)	
Not sure	24,135(55.3)	626 (2.6)	23,509 (97.4)	
Staple food [cases (%)]	43,672			0.007^*^
Primarily refined grain	17,767(40.7)	383 (2.2)	17,384 (97.8)	
Coarse and refined grain	20,012(45.8)	571 (2.9)	19,441 (97.1)	
Primarily coarse grain	2816(6.5)	64 (2.3)	2752 (97.7)	
Not sure	3077(7.0)	62 (2.0)	3015 (98.0)	
Milk consumption [cases (%)]	43,672			< 0.001^*^
No	15,406(35.3)	399 (2.6)	15,007 (97.4)	
1–2 times/week	19,345(44.3)	446 (2.3)	18,899 (97.7)	
3–5 times/week	6200(14.2)	164 (2.6)	6036 (97.4)	
Every day	2721(6.2)	71 (2.6)	2650 (97.4)	
Egg consumption [cases (%)]	43,672			< 0.001^*^
No	1811(4.1)	41 (2.3)	1770 (97.7)	
1–2 times/week	17,091(39.1)	369 (2.2)	16,722 (97.8)	
3–5 times/week	17,050(39.0)	396 (2.3)	16,654 (97.7)	
Every day	7720(17.7)	274 (3.5)	7446 (96.5)	
Bean/bean product consumption [cases (%)]	43,672			0.006^*^
No	2013(4.6)	49 (2.4)	1964 (97.6)	
1–2 times/week	26,361(60.4)	595 (2.3)	25,766 (97.7)	
3–5 times/week	13,522(31.0)	383 (2.8)	13,139 (97.2)	
Every day	1776(4.1)	53 (3.0)	1723 (97.0)	
Fruit consumption [cases (%)]	43,672			0.366
No	1948(4.5)	58 (3.0)	1890 (97.0)	
1–2 times/week	19,033(43.6)	431 (2.3)	18,602 (97.7)	
3–5 times/week	16,863(38.6)	426 (2.5)	16,437 (97.5)	
Every day	5828(13.3)	165 (2.8)	5663 (97.2)	
Vegetable consumption [cases (%)]	43,672			0.014^*^
< 100 g	5550(12.7)	138 (2.5)	5412 (97.5)	
100–199 g	24,617(56.4)	580 (2.4)	24,037 (97.6)	
200–499 g	11,773(27.0)	309 (2.6)	11,464 (97.4)	
≥ 500 g	1732(4.0)	53 (3.1)	1679 (96.9)	
Meat consumption [cases (%)]	43,672			0.024^*^
< 50 g	12,849(29.4)	329 (2.6)	12,520 (97.4)	
50–100 g	25,140(57.6)	642 (2.6)	24,498 (97.4)	
101–250 g	5337(12.2)	98 (1.8)	5239 (98.2)	
> 250 g	346(0.8)	11 (3.2)	335 (96.8)	
Animal viscera consumption [cases (%)]	43,672			< 0.001^*^
No	15,376(35.2)	307 (2.0)	15,069 (98.0)	
1–2 times/week	27,177(62.2)	741 (2.7)	26,436 (97.3)	
≥ 3 times/week	1119(2.6)	32 (2.9)	1087 (97.1)	
Fish consumption [cases (%)]	43,672			0.998
No	2961(6.8)	86 (2.9)	2875 (97.1)	
1–2 times/week	29,444(67.4)	716 (2.4)	28,728 (97.6)	
≥ 3 times/week	11,267(25.8)	278 (2.5)	10,989 (97.5)	
Coffee consumption [cases (%)]	43,672			0.244
No	33,512(76.7)	862 (2.6)	32,650 (97.4)	
1–2 times/week	8736(20.0)	181 (2.1)	8555 (97.9)	
3–5 times/week	1043(2.4)	29 (2.8)	1014 (97.2)	
Every day	381(0.9)	8 (2.1)	373 (97.9)	
Juice consumption [cases (%)]	43,672			0.025^*^
No	30,283(69.3)	794 (2.6)	29,489 (97.4)	
1–2 times/week	12,720(29.1)	268 (2.1)	12,452 (97.9)	
3–5 times/week	598(1.4)	16 (2.7)	582 (97.3)	
Every day	71(0.2)	2 (2.8)	69 (97.2)	
Smoking [cases (%)]	43,672			0.002^*^
No	30,051(68.8)	703 (2.3)	29,348 (97.7)	
Yes	10,716(24.5)	317 (3.0)	10,399 (97.0)	
Quit smoking	1870(4.3)	46 (2.5)	1824 (97.5)	
Passive smoking	1035(2.4)	14 (1.4)	1021 (98.6)	
Alcohol drinking [cases (%)]	43,672			< 0.001^*^
Never	38,315(87.7)	952 (2.5)	37,363 (97.5)	
Yes (> 1 day/week)	3372(7.7)	68 (2.0)	3304 (98.0)	
Quit drinking (at least 1 year)	1985(4.5)	60 (3.0)	1925 (97.0)	
Physical exercises [cases (%)]	43,672			< 0.001^*^
< 1 times/week	11,165(25.6)	242 (2.2)	10,923 (97.8)	
1–2 times/week	7391(16.9)	143 (1.9)	7248 (98.1)	
3–5 times/week	13,788(31.6)	321 (2.3)	13,467 (97.7)	
> 5 times/week	11,328(25.9)	374 (3.3)	10,954 (96.7)	

*Abbreviations:* *AMD* Age-Related Macular Degeneration, *SD* Standard Deviation, *BMI* Body Mass Index, *FBS* Fasting Blood Sugar, *HDL* High-Density Lipoprotein Cholesterol, *LDL* Low-Density Lipoprotein Cholesterol. *P < 0.05

According to Major Figures on 2020 Population Census of China [13], the age- and gender-standardized frequencies of early AMD in Hunan Province were calculated (Supplemental Table 3). The gender-standardized frequency of early AMD was 2.5% among the people aged 50 or above, which is almost the same as before standardization. And the age-standardized frequency was 6.6% among the people aged 60 or above, obviously up-regulated than before standardization.

**Fig2:**
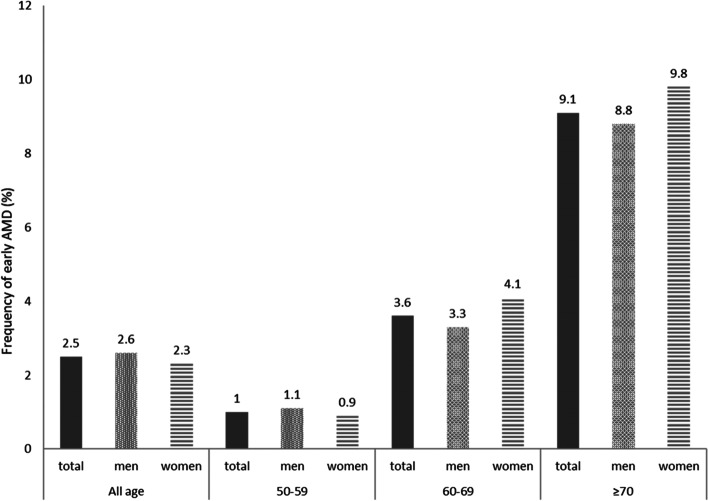
The distribution of early AMD by gender among the 43,672 participants in the whole cohort and different age groups (**, *p* < 0.01; ns: no significant difference.)

As shown in Fig. 2, the frequency of early AMD was significantly higher in men than in women in the whole cohort (*p* < 0.05) and in the 50–59 years old group (*p* > 0.05), but was higher in women than in men in the other two age groups (*p* > 0.05).

### Associations of early AMD with lifestyle and dietary characteristics

The associations of early AMD with lifestyle, clinical, and dietary characteristics of 43,672 study participants of ≥ 50 years old who underwent health examination during 2017–2019 are presented in Table 1. Repeated questionnaire assessment was conducted on 1675 study participants, and the results demonstrated a good reproducibility of the questionnaire (Supplemental Table 1). Univariate model demonstrated that age, gender, BMI, lymphocyte count, fasting blood glucose, blood lipids (cholesterol, triglyceride, and low-density lipoprotein), dietary pattern, salt intake, consumptions of milk, eggs, bean products, vegetables, meat, and animal viscera as well as smoking, alcohol drinking, physical exercises were associated with the risk of early AMD. These factors were included into multivariate logistic regression analysis after multiple collinear analysis and excluding variables with VIF value more than 10 (cholesterol) (Supplemental Table 2). BMI, Salt intake and smoking increased the risk of early AMD (*p* < 0.05), whereas meat consumption and alcohol consumption decreased the risk (both *p* < 0.05) (Table 2). In order to accurately determine the relationship between salt intake and AMD, we conducted subgroup analysis, interaction analysis and covariate screening. The results (Table 3) showed that the increase of salt intake was associated with the increase of the frequency of AMD in every stratified group including age, gender, being a smoker, the history of DM, hypertension and CKD. And we also found that age, gender, the history of hypertension had significant interaction effects on salt intake and AMD.

**Table 2:** Multivariable logistic regression analysis on the associations of early AMD with dietary characteristics of 43,672 participants of ≥ 50 years old who underwent health examination during 2017–2019

		Odds ration	95% CI		*P* value
			lower	upper	
Step 1	Age	1.10	1.09	1.10	.000
	Constant	.000			.000
Step 2	Age	1.10	1.10	1.11	.000
	Salt intake	1.61	1.54	1.68	.000
	Constant	.000			.000
Step 3	Age	1.10	1.10	1.11	.000
	Meat	.90	.81	.99	.033
	Salt intake	1.61	1.54	1.68	.000
	Constant	.000			.000
Step 4	Age	1.10	1.10	1.11	.000
	Meat	.90	.81	.99	.033
	Salt intake	1.61	1.54	1.68	.000
	Smoking	1.12	1.11	1.18	.034
	Constant	.000			.000
Step 5	Age	1.10	1.10	1.11	.000
	Meat	.90	.90	.99	.033
	Salt intake	1.61	1.54	1.68	.000
	BMI	1.02	1.01	1.05	.016
	Smoking	1.12	1.11	1.18	.034
	Constant	.000			.000
Step 6	Age	1.10	1.10	1.11	.000
	Meat	.90	.81	.99	.033
	Salt intake	1.61	1.54	1.68	.000
	BMI	1.02	1.01	1.05	.016
	Smoking	1.12	1.11	1.18	.034
	Alcohol drinking	0.93	0.91	0.97	.036
	Constant	.000			.000

*Abbreviations:* *BMI* Body Mass Index

**Table 3:** Stratified analysis of the association of salt intake on the risk of age-related macular degeneration

Group	**OR (95% Cl)**	***P***	*P* value for interaction
Gender			0.000*(1.036,1.062)
Men	1.45 (1.37,1.53)	0.000	
Women	1.59 (1.49,1.69)	0.000	
Age (years)			0.000*(1.12,1.13)
< 60	1.11 (1.05,1.17)	0.000	
60–69	1.69 (1.58,1.81)	0.000	
≥ 70	6.46 (5.12,8.14)	0.000	
DM			0.055(0.93,1.04)
NO	1.68 (1.56,1.81)	0.000	
Yes	1.35 (1.23,1.48)	0.000	
Hypertension			0.000*(1.01,1.03)
NO	1.11 (1.08,1.26)	0.000	
Yes	1.50 (1.35,1.66)	0.000	
CKD			0.078(0.92,1.03)
NO	1.51 (1.39,1.64)	0.000	
Yes	1.44 (1.35,1.55)	0.000	
Smoker			0.17(0.96,1.02)
NO	1.45 (1.39,1.52)	0.000	
Former	1.66 (1.40,1.97)	0.000	
Current	1.54 (1.09,2.18)	0.015	

*Abbreviations:* *DM* Diabetes Mellitus, *CKD* Chronic Kidney Disease *P < 0.05

## Discussion

In the present study, we estimated the frequency of AMD among people who underwent health examination in Hunan, China and determined the relationship between dietary pattern and the risk of AMD. Among the 43,821 study participants who underwent health examination, 1229 (2.80%) had AMD including 1080 (2.5%) early AMD and 149 (0.34%) late AMD. The frequency of early AMD was slightly higher in men than in women (2.6% vs. 2.3%). the frequency of early AMD was increased gradually with age in both genders. Multivariate logistic regression analysis demonstrated that BMI, salt intake and smoking increased the risk of early AMD, while meat consumption and alcohol drinking decreased the risk of early AMD.

According to the global estimation of the prevalence of AMD [1], early- and late-stage AMD were the most common in European populations, with prevalence of 11.20% and 0.50%; early AMD was rare in Asian populations, with a prevalence of 6.81%. At present, only four studies on the prevalence of AMD had been conducted in China, with the prevalence of early AMD ranging from 1.4% to 40.4% [14–17]. Of the four studies, two had been conducted 10 years ago [14, 15], two had been conducted among elderly populations in rural areas in Middle [15] and South China [16], one had been conducted among elderly populations in urban areas in East China [17]. These studies covered only limited populations in limited areas of China, with a wide time span. The differences in the aging trends of Chinese populations cannot be ignored. Currently, no report on the prevalence of AMD of the entire Chinese population has been published. Our study on the prevalence of AMD among participants who underwent health examination may enrich the database of AMD in China.

Age was confirmed to be a risk factor of AMD. While the association between gender and the risk of AMD is under debate. Our data demonstrated that the frequency of early AMD was significantly lower in women than in men among 43,672 participants of ≥ 50 years old who underwent health examination. However, in multivariate logistic regression gender was not the risk factor of the early AMD analysis, which was in consistent with the results from many previous studies [1, 18, 19].

The association between dietary pattern and diseases has been widely reported. Unhealthy dietary patterns, such as high fat and high red meat dietary patterns, are known to affect the development of AMD among genetic susceptible populations [20]. Healthy dietary patterns, such as Mediterranean dietary pattern [21], may provide antioxidant substances to the body and delay the occurrence of age-related diseases. At present, most studies on the direct influence of dietary pattern on AMD were conducted in European and American countries. Chiu et al. [22] used principal component analysis to analyze the food consumption data collected with the food frequency questionnaire (FFQ) released by the AREDS group and classified the dietary pattern as western and eastern dietary patterns. Through cross-sectional study, they found that the western dietary pattern, which was mainly composed of high-fat milk products, butter or margarine, gravy, processed food, eggs, sweeties, sport drinks, and refined food, increased the risk of AMD (OR = 3.70, 95% CI = 2.31–5.92, *p* < 0.0001); on the contrary, the eastern dietary pattern, which was mainly composed of vegetables, beans, rice, whole grains, fruits, tomato, vegetables with green leaves, low-fat milk products, fish and seafood, decreased the risk of AMD (OR = 0.38, 95% CI = 0.27–0.54, *p* < 0.0001). In China, diverse dietary patterns with complex compositions are adopted, and their associations with AMD have not been well studied. In the present study, we used the National Unified Physical Examination Questionnaire to collect dietary and lifestyle data and analyzed their associations with AMD. We found that excessive salt intake (OR = 1.60, 95% CI = 1.53–1.67, *p* < 0.001) and smoking (OR = 1.13, 95% CI = 1.11–1.18, p = 0.024) increased the risk of early AMD.

High-salt intake has been widely proved to be associated with the risks of many diseases, such as hypertension [23], angiocardiopathy [24], and gastric cancer [25]. According to the 2015 global disease burden study [26], high-salt intake was listed as one of the top ten largest contributors to global disability-adjusted life years. In the Diet, Nutrition, and the Prevention of Chronic Diseases: Report of a Joint WHO/FAO Expert Consultation conducted by World Health Organization (WHO) and Food and Agricultural Organization (FAO), the recommended salt intake for Chinese was lower than 5 g/day. The Chinese Nutrition Society suggested that the salt intake of Chinese should be no more than 6 g/day. Yu et al. [27] reported that the average salt intake of Chinese was 9.6 ± 0.3 g/day. Our data demonstrated that the average salt intake was 8.39 ± 0.68 g/day in participants without AMD and 9.26 ± 0.56 g/day in those with early AMD. Yang et al. [10] analyzed the salt intake data from Hunan participants of > 65 years old who underwent health examination and reported an average salt intake of 8.15 ± 1.89 g/day. These data indicate that the salt intake of elderly populations in Hunan, China is much higher than the recommended level, severely affecting the health of the eyes and the body. The high salt intake of people in Hunan is related to their eating habits. This area likes to eat salt-pickled meat, fish, etc. The major source of salt (about 70%) comes from processed food. The salt intake is increased rapidly along with the increased consumption of processed food. In the cell experiment, increased salt intake was reported to be an environmental risk factor of AMD [23]. High-salt intake may induce cellular dysfunctions, such as DNA double-strand break, DNA and protein oxidation, structural and functional impairment of mitochondria, cytoskeleton changes, and apoptotic cell death. It may increase the risk of AMD through inducing oxidative stress of RPE cells. The damage of high-salt intake to the retina varied among individuals. In a study on patients with refractory hypertension, it was reported that high-salt intake thickened the arteriolar wall in the retina [28]. In salt-sensitive rats, high-salt intake led to retina arteriolar spasm and ischemia [29].

Experimental study further proved that the high extracellular permeation pressure induced by high-salt intake stimulated RPE cells to express vascular endothelial growth factor (VEGF), basic fibroblast growth factor (bFGF), placental growth factor (PlGF), and Heparin-binding epidermal growth factor (HB-EGF) [30–32]. The transcription activity of hypoxia-inducible factor-1 (HIF-1) and nuclear factor of activated T cell-5 (NFAT5), which is important in the regulation of VEGF, is also affected by the high-salt permeation pressure [30]. The increased plasma osmotic pressure induced by high-salt intake may stimulate regional inflammation in RPE and production of angiogenic factors, leading to the progression of AMD [32].

It is the first time to confirm that high salt intake increased the risk of AMD in the population study. The subgroup analysis found that high salt intake had statistical effect both in non-CKD group and in CKD group in our study. And we also observed the similar results both in non-DM group and in DM group. In addition, we observed some interesting phenomena. Although interactive analysis showed that there was an interaction between hypertension and salt intake. However, stratified analysis showed that high salt intake may have a greater impact on patients without hypertension (OR = 1.62, 95% CI = 1.45–1.81, *p* < 0.01). In the no hypertension group, the increased salt intake was associated with the higher risk of early AMD, while the corresponding values were not significant for hypertension person (OR = 1.00, 95% CI = 0.99–1.03, *p* = 0.083).

In subgroup analysis, our study still showed that high-salt intake had the statistical significance on the frequency of AMD among no-smokers, before-smokers, and current-smokers. Interactive analysis showed that there was no interaction between smoking and salt intake. Therefore, it also confirmed that smoking is an independent risk factor for AMD. Interestingly, in the present study, we found that the meat consumption decreased the risk of early AMD (OR = 0.89, 95% CI = 0.80–0.99, *p* = 0.037). This result was not in consistent with those from Chiu et al. [24]. They found that the western dietary pattern, which was mainly composed of meat, fat, and butter, increased the risk of AMD, but the relationship between meat and the risk of AMD was not investigated. The ketogenic diet was reported to increase β-hydroxybutyrate (β-HB) level, which may prevent or alleviate symptoms of age-related diseases, exert antiaging effect [33], and prolong the lifespan [34, 35]. In mammals, β-HB may down-regulate senescence-associated secretory phenotype (SASP) and retard the senescence of vascular cells [36]. The ketogenic diet may also alleviate symptoms of Alzheimer disease, an age-related neurodegenerative disease [37, 38]. The dietary pattern adopted by elderly populations in Hunan, China is high-fat and meat-dominant with limited carbohydrates, which is somewhat like to the characteristics of ketogenic diet. Our observation needs to be validated in a prospective cohort study.

Moreover, it should be noted that the confounding effect of lifestyle factors on the frequency of AMD cannot be completely excluded. In addition, the participants who underwent health examination in the present study could not represent general populations, especially those in poverty areas and rural areas with limited access to health examination. The proportion of such participants was low in our cohort, indicating a sampling bias in the present study. In addition, selection bias could not be avoided due to the inclusion of various examinations, such as fundus examination, fundus photography, OCT, and MSI, for the diagnosis of AMD in the present study, affecting the estimation of the frequency of AMD. Although these limitations existed, we still observed associations between unhealthy dietary patterns and the risk of early AMD.

## Conclusion

In Hunan China**,** there was a high frequency of early AMD detected through health examination over the 50 years old. And high-salt intake is associated with the risk of early AMD. A meat-dominant dietary pattern may decrease the risk of early AMD. Modulating the dietary pattern and reducing the salt intake as an AMD prevention strategy warrant further study.

## Supplementary Information

**Additional file 1: Table 1. **Cohen's kappa comparing food intake at baseline with a repeat questionnaire within 1-2 years (*n* =1675).

**Additional file 2: Table 2-1.** Multiple collinearity analysis of independent variables on the associations of early AMD with biochemical characteristics of 43,672 participants of ≥50 years old who underwent health examination during 2017-2019. **Table 2-2.** Multiple collinearity analysis of independent variables exclude the variable Cholesterol (VIF＞10).

**Additional file 3: Table 3. **Standardized frequency of AMD in Hunan people.

**Additional file 4: Table 4.** Multivariable logistic regression analysis on the associations of late AMD with dietary characteristics of study participants of ≥50 years old who underwent health examination during 2017-2019.

## Data Availability

All data generated or analysed during this study are included in this published article. The datasets generated and/or analysed during the current study are not publicly available due ethical concerns but are available from the corresponding author on reasonable request.

## Abbreviations

AMD: Age-related macular degeneration
THcy: Total homocysteine
HHcy: Hyperhomocysteinemia
CKD: Chronic kidney disease
DM: Diabetes mellitus
NAMD: Non-AMD
