# Supplementary material for: The frequency of early age-related macular degeneration and its relationship with dietary pattern in Hunan, China: a cross-sectional study
Source: BMC Ophthalmol. 2022 Jul 27;22:324. doi: 10.1186/s12886-022-02549-x (PMC9327240; doi:10.1186/s12886-022-02549-x)
Supplement: Supplementary file 1 — Additional file 1: Table 1. Cohen's kappa comparing food intake at baseline with a repeat questionnaire within 1-2 years (n =1675). [file 12886_2022_2549_MOESM1_ESM.docx]

**Supplemental Table 1.** Cohen's kappa comparing food intake at baseline with a repeat questionnaire within 1-2 years (n =1675).

| Variants | kappa | Sig |
| --- | --- | --- |
| Dietary pattern | 0.94 | 0.000* |
| Milk | 0.91 | 0.000* |
| Egg | 0.92 | 0.000* |
| Bean products | 0.83 | 0.000* |
| Vegetables | 0.76 | 0.000* |
| Meat | 0.81 | 0.000* |
| Animal viscera | 0.85 | 0.000* |
| Juice | 0.81 | 0.000* |
| Smoking | 0.84 | 0.000* |
| Alcohol drinking | 0.83 | 0.000* |
| Physical exercises | 0.88 | 0.000* |

**P* < 0.05
