# Supplementary material for: The frequency of early age-related macular degeneration and its relationship with dietary pattern in Hunan, China: a cross-sectional study
Source: BMC Ophthalmol. 2022 Jul 27;22:324. doi: 10.1186/s12886-022-02549-x (PMC9327240; doi:10.1186/s12886-022-02549-x)
Supplement: Supplementary file 2 — Additional file 2: Table 2-1. Multiple collinearity analysis of independent variables on the associations of early AMD with biochemical characteristics of 43,672 participants of ≥50 years old who underwent health examination during 2017-2019. Table 2-2. Multiple collinearity analysis of independent variables exclude the variable Cholesterol (VIF＞10). [file 12886_2022_2549_MOESM2_ESM.docx]

**Supplemental table 2-1.** Multiple collinearity analysis of independent variables on the associations of early AMD with biochemical characteristics of 43,672 participants of ≥50 years old who underwent health examination during 2017-2019.

| Variable | Tolerance | VIF |
| --- | --- | --- |
| Age | .824 | 1.213 |
| Gender | .616 | 1.622 |
| Education | .805 | 1.242 |
| BMI | .835 | 1.197 |
| Lymphocyte count | .953 | 1.049 |
| FBS | .920 | 1.087 |
| Cholesterol | .095 | 10.546 * |
| Triglyceride | .238 | 4.208 |
| LDL | .100 | 9.999 |
| Salt intake | .980 | 1.020 |
| Dietary pattern | .959 | 1.042 |
| Staple food | .992 | 1.008 |
| Milk | .785 | 1.273 |
| Egg | .777 | 1.286 |
| Bean products | .883 | 1.133 |
| Vegetables | .858 | 1.165 |
| Meat | .878 | 1.139 |
| Animal viscera | .822 | 1.217 |
| Juice | .932 | 1.072 |
| Smoking | .811 | 1.233 |
| Alcohol drinking | .853 | 1.173 |
| Physical exercises | .850 | 1.177 |

Abbreviations: BMI body mass index; FBS fasting blood glucose; LDL low density lipoprotein cholesterol.

*VIF＞10, The variable should be removed.

**Supplemental table 2-2.** Multiple collinearity analysis of independent variables exclude the variable Cholesterol (VIF＞10)

| Variable | Tolerance | VIF |
| --- | --- | --- |
| Age | .824 | 1.213 |
| Gender | .650 | 1.539 |
| Education | .806 | 1.241 |
| BMI | .862 | 1.160 |
| Lymphocyte count | .954 | 1.048 |
| FBS | .920 | 1.087 |
| Triglyceride | .836 | 1.196 |
| LDL | .907 | 1.102 |
| Salt intake | .981 | 1.019 |
| Dietary pattern | .960 | 1.042 |
| Staple food | .906 | 1.103 |
| Milk | .785 | 1.273 |
| Egg | .778 | 1.286 |
| Bean products | .883 | 1.132 |
| Vegetables | .858 | 1.165 |
| Meat | .878 | 1.139 |
| Animal viscera | .822 | 1.217 |
| Juice | .933 | 1.072 |
| Smoking | .811 | 1.232 |
| Alcohol drinking | .858 | 1.166 |
| Physical exercises | .850 | 1.176 |
