# Supplementary material for: The frequency of early age-related macular degeneration and its relationship with dietary pattern in Hunan, China: a cross-sectional study
Source: BMC Ophthalmol. 2022 Jul 27;22:324. doi: 10.1186/s12886-022-02549-x (PMC9327240; doi:10.1186/s12886-022-02549-x)
Supplement: Supplementary file 3 — Additional file 3: Table 3. Standardized frequency of AMD in Hunan people. [file 12886_2022_2549_MOESM3_ESM.docx]

**Supplemental table 3.** Standardized frequency of AMD in Hunan people

Gender-standardized frequency of AMD in Hunan people over 50 years old

| population composition in Hunan^#^ (*100 000) | | Frequency | Standardized frequence (per 100 000) | Standardized frequency |
| --- | --- | --- | --- | --- |
| men | 340.0 | 2.6% | 8.84 |  |
| women | 324.5 | 2.3% | 7.46 |  |
| total | 664.5 | 2.5% | 16.3 | 2.5% |

^#^ According to Major Figures on 2020 Population Census of China

Age-standardized frequency of AMD in Hunan people over 60 years

| population composition in Hunan^#^ (*100 000) | | Frequency | Standardized frequence (per 100 000) | Standardized frequency |
| --- | --- | --- | --- | --- |
| 60-65 years | 33.7 | 2.3% | 0.78 |  |
| ≥65 years | 98.4 | 8.1% | 7.97 |  |
| total | 132.1 | 5.4% | 8.75 | 6.6% |

^#^ According to Major Figures on 2020 Population Census of China
