# Supplementary material for: The frequency of early age-related macular degeneration and its relationship with dietary pattern in Hunan, China: a cross-sectional study
Source: BMC Ophthalmol. 2022 Jul 27;22:324. doi: 10.1186/s12886-022-02549-x (PMC9327240; doi:10.1186/s12886-022-02549-x)
Supplement: Supplementary file 4 — Additional file 4: Table 4. Multivariable logistic regression analysis on the associations of late AMD with dietary characteristics of study participants of ≥50 years old who underwent health examination during 2017-2019. [file 12886_2022_2549_MOESM4_ESM.docx]

**Supplemental table 4.** Multivariable logistic regression analysis on the associations of late AMD with dietary characteristics of study participants of ≥50 years old who underwent health examination during 2017-2019

|  | | Odds ration | 95% CI | | *P* value |
| --- | --- | --- | --- | --- | --- |
|  |  |  | lower | upper |  |
| Step 1 | Age | 1.08 | 1.06 | 1.11 | .000 |
|  | Constant | .000 |  |  | .000 |
| Step 2 | Age | 1.08 | 1.06 | 1.11 | .000 |
|  | Salt intake | 1.11 | 1.050 | 1.17 | .000 |
|  | Constant | .000 |  |  | .000 |
| Step 3 | Gender | 2.36 | 1.49 | 3.73 | .000 |
|  | Age | 1.09 | 1.07 | 1.12 | .000 |
|  | Salt intake | 1.11 | 1.05 | 1.18 | .000 |
|  | Constant | .000 |  |  | .000 |
| Step 4 | Gender | 2.02 | 1.26 | 3.22 | .003 |
|  | Age | 1.09 | 1.07 | 1.12 | .000 |
|  | Education level | .68 | .53 | .88 | .003 |
|  | Salt intake | 1.12 | 1.06 | 1.18 | .000 |
|  | Constant | .000 |  |  | .000 |
| Step 5 | Gender | 2.48 | 1.51 | 4.08 | .000 |
|  | Age | 1.09 | 1.07 | 1.12 | .000 |
|  | Waist-to-hip ratio | 1.04 | 1.01 | 1.06 | .011 |
|  | Education level | .70 | .54 | .90 | .005 |
|  | Salt intake | 1.10 | 1.05 | 1.17 | .000 |
|  | Constant | .000 |  |  | .000 |
| Step 6 | Gender | 2.29 | 1.39 | 3.78 | .001 |
|  | Age | 1.09 | 1.07 | 1.12 | .000 |
|  | Waist-to-hip ratio | 1.03 | 1.01 | 1.06 | .012 |
|  | LDL | 1.34 | 1.05 | 1.72 | .020 |
|  | Education level | .69 | .54 | .90 | .005 |
|  | Salt intake | 1.10 | 1.04 | 1.16 | .001 |
|  | Constant | .000 |  |  | .000 |

Abbreviations: LDL low-density lipoprotein cholesterol.
